# Drug Usability Survey (DUS) of Gabapentinoid and Its Combinations Among Indian Patients With Neuropathic Pain: Results From a Real-World, Multicenter, Retrospective Survey at Neurology Clinics

**DOI:** 10.7759/cureus.79722

**Published:** 2025-02-26

**Authors:** Puneet Aggarwal, Pashupati Nath Mishra, VN Mathur, Kiran C Velivela, Siraj Khan, Prashant Deshmukh, Maneesha Khalse, Kamlesh Patel

**Affiliations:** 1 Neurology, Max Super Specialty Hospital,, New Delhi, IND; 2 Neurosurgery, KH Advanced Brain and Spine Centre, Apollo KH Hospital, Vellore, IND; 3 Neurology, Dr. VN Mathur Hospital, Hyderabad, IND; 4 Neurosurgery, NRI Academy of Sciences, Guntur, IND; 5 Neurology, Saboo Siddique Maternity and General Hospital, Mumbai, IND; 6 Medical Services, Medical Affairs Division, Lupin Limited, Mumbai, IND

**Keywords:** drug utilization survey (dus), gabapentin, gabapentinoids, india, neuropathic pain, non-opioid pain strategies, pain management, peripheral neuropathy, pregabalin

## Abstract

Introduction: Neuropathic pain of various etiology is the most commonly reported at primary clinics by patients. Patients experience moderate to severe chronic pain, impacting quality of life (QoL) and mood. The mainstay of the treatment includes gabapentinoid-based treatment to reduce pain severity and improve the QoL for the patients.

Methods: In a retrospective cross-sectional survey in India, the drug usability of gabapentinoid-based treatment in various neuropathic pain was studied. This included data collection from various neurological clinics across India that considered patient demographics, comorbidities, type of neuropathies, the percentage of patients receiving gabapentinoid-based treatment, the share of diabetic patients and diabetic neuropathy, and the severity of pain reported by patients.

Results: The cross-sectional survey was conducted at 51 neurology clinics involving 2,251 patients. Patients presented with neuropathic pain of various etiologies, of which diabetic neuropathy was the most prevalent condition. Among the patients, 59.30% (1,252) consulted the neurologist for the first time, whereas 40.70% (860) of patients visited the clinic for follow-up. Neurologists prescribed gabapentinoid-based combination treatment as the main preferred treatment. Duloxetine, a selective serotonin and norepinephrine reuptake inhibitor (SSNRI) antidepressant, and nortriptyline, a tricyclic antidepressant (TCA), were the most preferred agents used in combination with pregabalin and gabapentin. Patients who visited for follow-up reported pain reduction and improved QoL with the treatment provided by the neurologists.

Conclusion: Gabapentinoid-based treatments combined with TCA and SSNRI are useful and well-accepted treatment modalities by neurologists in painful neuropathies. Gabapentinoids are non-opioids with no risk of abuse and addiction and were considered the first line of therapy for various types of neuropathic pain.

## Introduction

Neuropathic pain is the most frequent health condition encountered by clinicians in daily practice. It is a chronic condition known to affect patients’ quality of life (QoL) and social, economic, and psychological well-being [1]. Globally, an estimated 7% to 10% of adults suffer from neuropathic pain, with a reported prevalence of peripheral neuropathy of 2.4%, which increases with age to an estimated 8% in those older than 55 years [2,3]. This condition has several underlying causes and has varied clinical presentations [4]. Neuropathic pain results from the nerves' demyelination caused by various factors, of which oxidative stress is a contributor [5]. Apart from stabbing pain, the patient experiences numbness, tingling, burning sensation, etc., and often experiences weakness in the hands, feet, and other body parts. Neuropathic pain, comprised of a range of heterogeneous conditions, is often severe and difficult to manage and affects the overall functioning and QoL in patients [6]. Among the various neuropathic pains, peripheral neuropathy is highly prevalent, is associated with many conditions, and requires treatment for the underlying cause. Diabetes is a significant risk factor, strongly associated with peripheral neuropathy [7,8]. Neuropathic pain and burning sensation cause severe patient discomfort and are the clinic's most reported symptoms [9]. Thus, alleviating pain symptoms and improving QoL in patients remains a primary goal of treatment apart from treating the underlying disorder/disease [10].

The neuropathic pain management treatment algorithm considers multidisciplinary conservative care and nonopioid medications such as tricyclic antidepressants (TCA), selective serotonin and norepinephrine reuptake inhibitor (SSNRI) antidepressants, gabapentinoids, topicals, and transdermal substances as the first-line therapy [11]. Combination therapy with the first-line medications with tramadol/tapentadol is recommended as second-line therapy. The third-line therapy includes serotonin-specific reuptake inhibitors/anticonvulsants/N-methyl-D-aspartate (NMDA) antagonists and interventional therapies. Neurostimulation is ranked the fourth-line treatment, whereas the treatment with low-dose opioids (no greater than 90 morphine equivalent units) is considered the fifth-line treatment option. The last option is the targeted drug delivery for patients with refractory pain [12]. With this extensive treatment algorithm being recommended for treating neuropathic pain, it is undoubtedly a matter of interest to assess the use of various interventions and if they have been used as per the recommended algorithm. A cross-sectional drug utilization survey (DUS) in a large patient cohort can provide insight into the usage pattern of the available pharmacological interventions in the primary healthcare setting. This paper presents the results of a DUS planned at neurology clinics across India to evaluate the prescription pattern in terms of choice of gabapentinoids as monotherapy or in combination with various types of neuropathic pain. Additionally, an analysis of patient demographics, related symptoms, associated comorbidities, and patient-reported QoL improvement was also carried out and is presented based on collected data. 

## Materials and methods

A non-interventional, retrospective survey was planned to evaluate the usability pattern of gabapentinoids and their combinations among Indian patients presenting with neuropathic pain. This survey was intended to collect data from the medical records of various primary healthcare clinics across India. An independent ethics committee, the Good Society For Ethical Research approved the study protocol before initiation (approval number: S/0010076/NE/2012). Before study site recruitment, a site feasibility was conducted to identify eligible clinical sites for participation. The primary criterion for site selection was the availability of medical records of the patients with neuropathic pain visiting the neurology clinic. The study proforma included the details of the patients above 18 years of age presenting neuropathic pain who have received or were initiated with gabapentinoid or its combination. Incomplete medical records and data on pregnant and lactating mothers were excluded. The primary outcomes considered were as follows: percentage of patients receiving gabapentinoids and their combinations and assessment of demographics, comorbidities, and type of neuropathies.

The secondary outcomes considered demographic parameters with various neuropathies and their association in the observed cohort. The intensity of pain was measured using a 0-10 numeric pain rating scale. No structured QoL questionnaire was considered; the improvement in QoL was assessed by asking if the patient felt any improvement in pain and activities. Patients answered either yes or no. 

The participating site needed to complete a customized paper-based case report form (patient report sheet) prepared for data collection. The study monitoring and data verification activities were contracted out to an independent clinical research organization (CRO), and the study was conducted according to applicable regulations, Good Clinical Practice (GCP) guidelines, and CRO’s standard operating procedures. The CRO was responsible for verifying the filled data from the site and confirming its accuracy and completeness as per the study protocol and authenticity. The data management was also outsourced for independent data entry and statistical analysis. 

Statistical methods

This was a real-world, retrospective, observational study; data collection of approximately 3000 patients from about 60 study sites across India was planned, and no specific sample size was considered. The statistical plan considered assessing the frequency distribution of various parameters such as demographic data, treatment data, and type of symptoms reported by the patients. The data were depicted in counts and/or in percentages. 

## Results

This cross-sectional survey was conducted between January 1, 2023, and January 24, 2024. A total of 2,251 patients' medical records were reviewed for data collection from 51 neurology clinics spread across India. Among the 2,251 patient records, there were 1,324 male and 818 female patients. The average age of patients was 51.4±13.34 years, with body weight ranging from 24 to 120 kg, averaging 67.22± 11.695 kg. Among the age distribution, 29.81% (659 patients) were between the ages of 18 and 44 years; 28.55% (631 patients) were in the age range of 45-54 years, and 24.88% (550 patients) were in the 55-64 years. The 65 years and older group was the smallest, with 16.74% (370 patients). 

**Figure 1:**
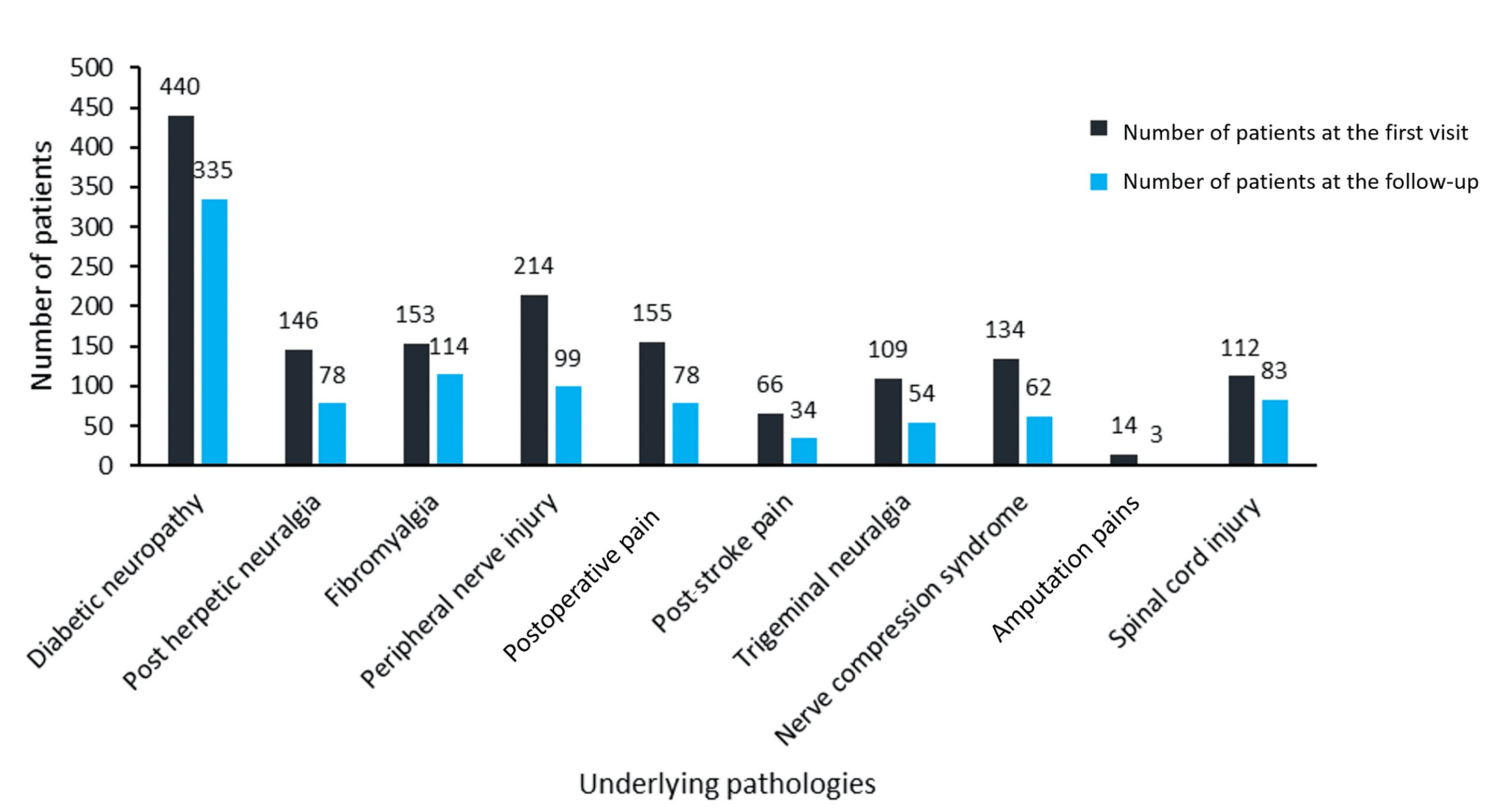
Types of neuropathic pain presented by patients (N=2483)

Among the comorbidities, diabetes mellitus (DM) alone was the most common condition present in 28.38% of the population (543 subjects), followed by hypertension alone in 19.86% (380 subjects). Hyperthyroidism was present in 5.91% (113 subjects), arthritis in 6.53% (125 subjects), and hyperlipidemia in 1.15% (22 subjects) as a single pathology, respectively. Among the multiple comorbid conditions, diabetes with hypertension was predominant in 15.58% (298 subjects) of the cohort. Patients sought treatment for various types of neuropathic pain (Figure 1).

**Figure 2:**
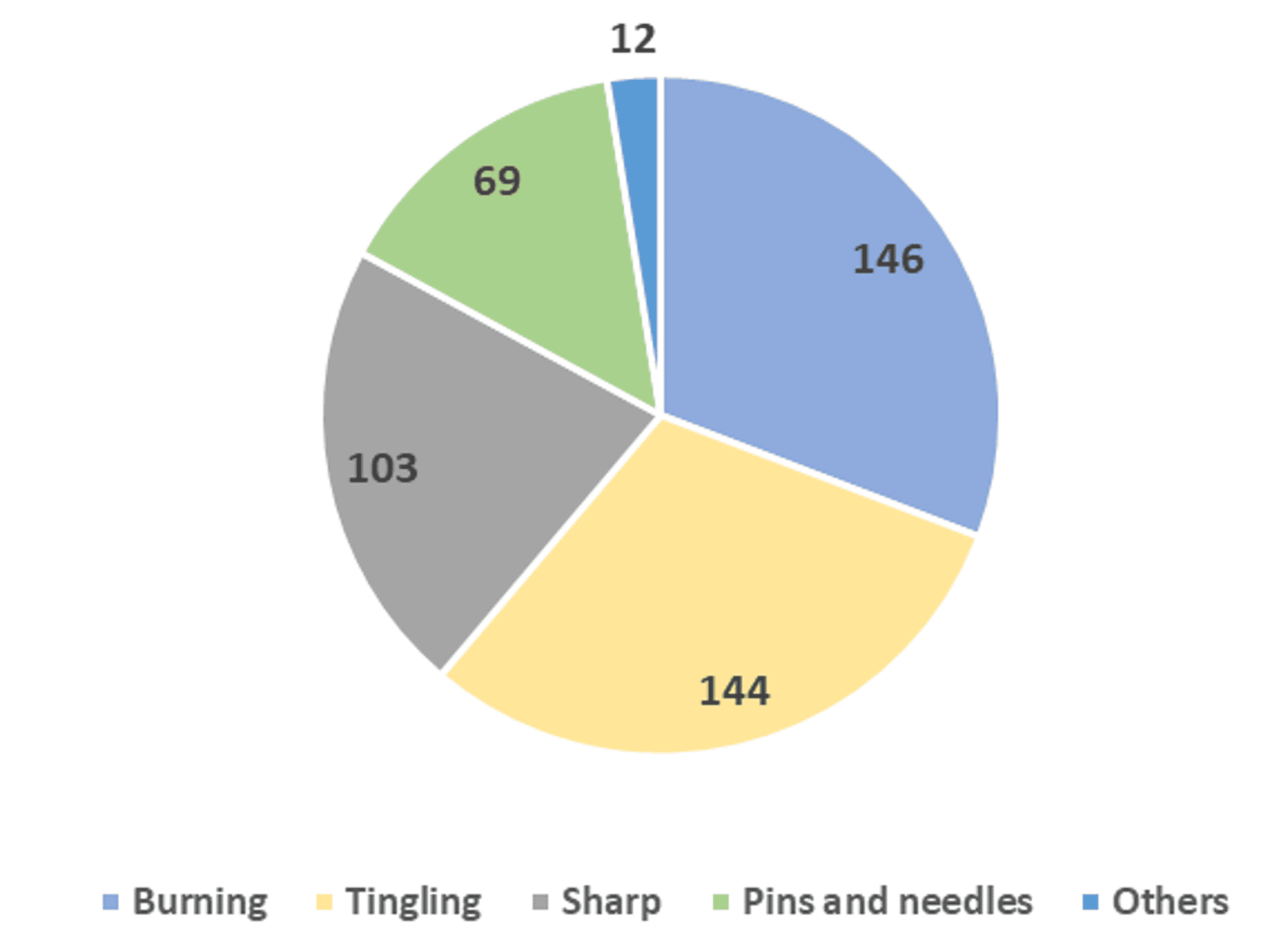
Type of pain symptoms reported by the patients (N=474)

Among the patients, only 0.04% (one patient) reported no pain, whereas 6.99% (145 patients) reported mild pain, 47.03% (975 patients) reported moderate pain, and 45.92% (952 patients) reported severe pain. Low back pain was reported by patients with diabetes (38.6%) and hypertension (33%). Patients with arthritis (31) had a common complaint of pain in the legs and hips. Among the 2,251 patients, 59.30% (1,252 patients) were first-time visitors to the clinic to seek consultation for their neuropathic pain, whereas 40.70% (860 patients) records were from the patients visiting the clinic for follow-up. Diabetic neuropathy (31.50%) remained the most prominent medical condition, seeking medical consultation among the cohort. Other notable medical conditions seeking treatment for neuropathic pain included peripheral nerve injury (12.40%), fibromyalgia (10.70%), and nerve compression syndrome (8.20%). A small percentage of patients sought treatment for post-herpetic neuralgia, postoperative pain, and trigeminal neuralgia. Low back pain was reported by 51.80% of patients, followed by circulation Pain at 35.50%. Pain in the legs and hips and other pain types were reported by 25.80% and 34.90% of patients, respectively. Pain in the neck and shoulder was reported by 4% of individuals. Diabetic neuropathy was the most common complaint (440 patients for the first visit) for which the patients sought consultation with the neurologists. The patients already receiving medicines for treating their neuropathic pain expressed the signs and symptoms of the pain, categorized as burning, tingling, sharp, pins and needles type, and others (Figure 2).

All patients could report the time when the pain felt most intense. Around 673 patients (28.3%) reported more pain during the daytime, whereas 727 patients (30.5%) experienced pain at night, and 215 patients (9.0%) reported pain in the evening; 52.10% of patients reported worsening pain while carrying out daily activities. The survey recorded the clinicians' prescription patterns. Clinicians prescribed gabapentinoids alone or in combination with SSNRI or TCA to treat various types of neuropathic pain (Table 1).

**Table 1:** Treatment received by the patients to treat various types of neuropathic pain (N=2841)

Types of pain		Pregabalin	Gabapentin	Pregabalin + Nortriptyline	Gabapentin + Nortriptyline	Pregabalin + Duloxetine
Diabetic neuropathy		199 (32%)	90 (30%)	217 (33%)	87 (25%)	269 (29%)
Post-herpetic neuralgia		59 (10%)	39 (13%)	71 (11%)	30 (8%)	89 (10%)
Fibromyalgia		69 (11%)	42 (14%)	46 (7%)	34 (10%)	96 (11%)
Peripheral nerve injury		71 (12%)	26 (9%)	73 (11%)	51 (14%)	155 (17%)
Postoperative pain		66 (11%)	19 (6%)	52 (8%)	37 (10%)	65 (7%)
Post-stroke pain		27 (4%)	13 (4%)	23 (3%)	19 (5%)	29 (3%)
Trigeminal neuralgia		32 (4%)	21 (7%)	43 (7%)	30 (8%)	60 (7%)
Nerve compression syndrome		41 (7%)	26 (9%)	58 (9%)	40 (11%)	59 (6%)
Amputation pains		5 (1%)	2 (1%)	5 (1%)	2 (1%)	6 (1%)
Spinal cord injury		28 (5%)	18 (6%)	50 (8%)	19 (5%)	75 (8%)
Others		16 (3%)	5 (2%)	22 (3%)	4 (1%)	11 (1%)

**Figure 3:**
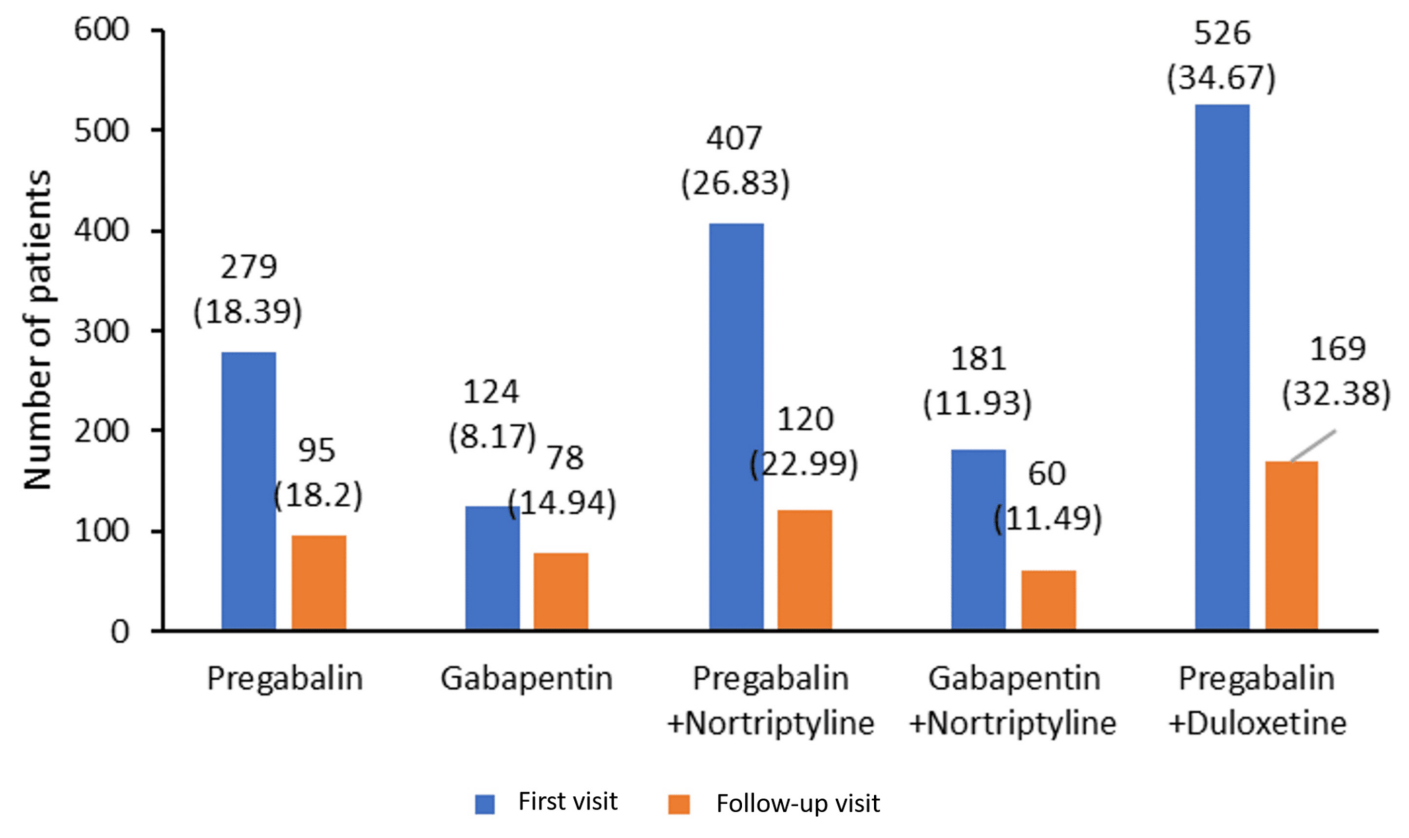
Treatment received by the patients (N=2039) Figures in the parentheses denote percentages (%)

The prescribed treatment for the patients who visited for the first time (first visit) and patients who visited for their follow-up visit was captured (Figure 3). Pregabalin with duloxetine was the predominant combination therapy recommended to the patients at the first visit as well as at the follow-up visit.

**Figure 4:**
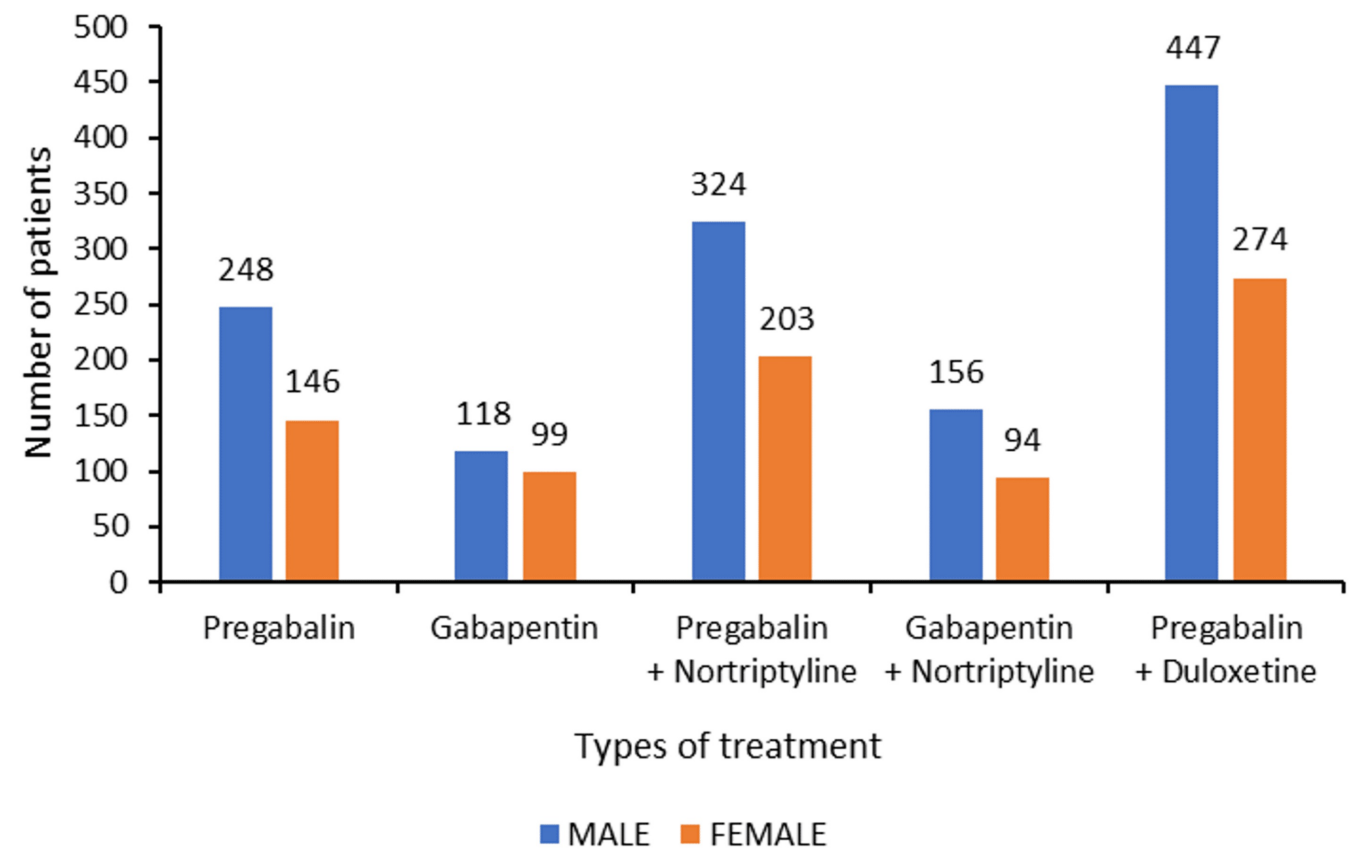
Gender-wise distribution of the treatment provided to the patients (N=2109)

There was no gender difference in the recommended treatment for male and female patients (Figure 4). 

**Figure 5:**
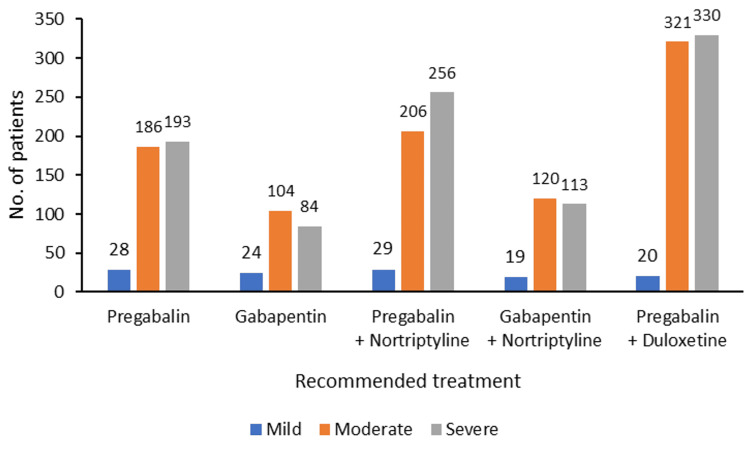
Pain severity and treatment received (N=2033)

The treating neurologists considered the severity of the patient's pain while choosing the treatment. Pregabalin + duloxetine was the intervention of choice for treating moderate to severe pain in the majority of the patients (Figure 5).

**Figure 6:**
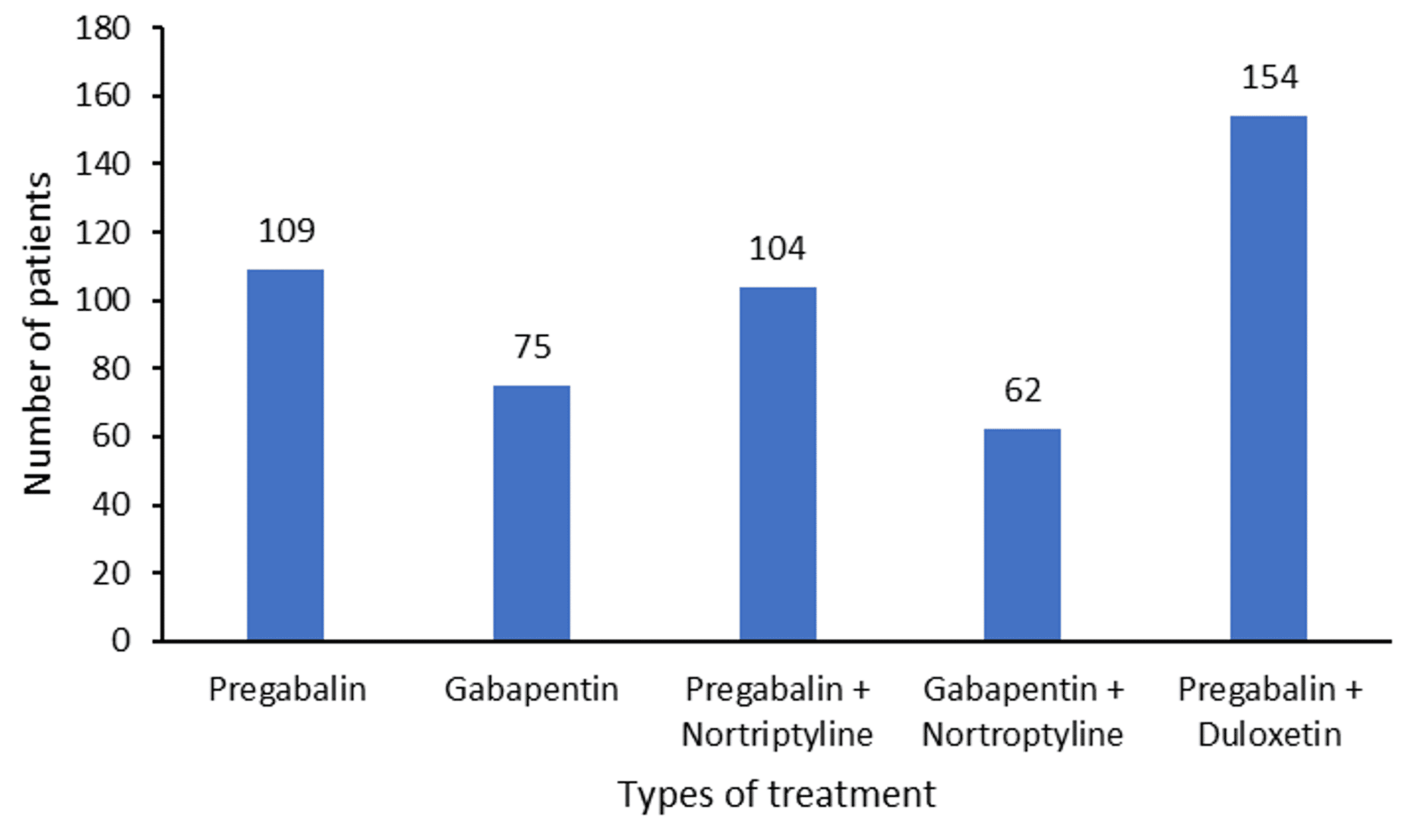
Patient-reported quality of life improvement (N=504)

Improvement following treatment was captured for the patients visiting for follow-up. Among these, pregabalin + nortriptyline demonstrated the highest improvement rate at 85.32%, pregabalin + duloxetine at 83.78%, gabapentin alone at 76.23%, and gabapentin + nortriptyline at 78.13%. In the case of follow-up visits, patients visited at different time intervals, ranging from week one to week four post their first visit. Of the patients (n=555) who visited for follow-up, post one week, two weeks, three weeks, and four weeks of their follow-up, all 50, 287, 125, and 93 patients, respectively, reported improvement from the baseline pain symptoms. Among these patients, 75.12% of patients on pregabalin + duloxetine, 79.48% on gabapentin + nortriptyline, 75.36% of patients on pregabalin + nortriptyline, and 69.44% of patients on gabapentin alone showed improvement in QoL with the medication provided for their neuropathic pain (Figure 6).

Low back pain was the most common complaint reported by 1,104 patients, followed by leg and hip pain in 738 patients. Pregabalin + duloxetine was the most prominently used treatment for pain with multiple etiologies.

In the patient follow-up cohort, a change in prescription from the recommended initial medication was observed. The change in prescription was prompted by the reported tolerance profile experienced by the patient was as follows: 53 of 151 (35%) patients receiving pregabalin, 10 of 70 (14.28%) patients receiving gabapentin, 29 of 118 (24.57%) patients receiving pregabalin + nortriptyline, 15 of 71 (21.12%) patients receiving gabapentin + nortriptyline, and 27 of 137 (19.70%) patients receiving pregabalin + duloxetine had a change of prescription at follow-up visits. The rest of the patients continued with the initial treatment.

The shift in prescription prompted by the lack of satisfactory efficacy among the patients from the follow-up cohort was as follows: 49 of 174 (28.16%) from pregabalin, 33 of 98 (33.67%) from gabapentin, 35 of 130 (26.92%) from pregabalin + nortriptyline, 24 of 79 (30.37%) from gabapentin + nortriptyline, and 68 of 193 (35.23%) from the pregabalin + duloxetine group. IPregabalin + duloxetine (1,104 of 2,251 (49.04%) patients) emerged as the most commonly prescribed combination for pain among the combination treatments.

## Discussion

Globally, neuropathic pain, caused by a lesion or disease affecting the somatosensory system, affects between 3% and 17% of the general population [12]. The reported prevalence of neuropathic pain in India is 7% to 8% in adults [13]. In the literature, the reported frequency of chronic neuropathic pain is higher in women (8%) than in men (5.7%) and more common in patients over 50 years old (8.9%) than in those under 50 years old (5.6%). The lower and upper limbs, lumbar spine, and neck have been the most frequently reported chronic neuropathic pain sites [14,15]. It has been reported that one-third of all community-based diabetic patients have painful neuropathy symptoms; it is also reported that peripheral diabetic neuropathy is more prevalent in patients with type 2 diabetes, women, and people of South Asian origin [16]. The most commonly reported conditions associated with neuropathic pain include postherpetic neuralgia, trigeminal neuralgia, painful radiculopathy, diabetic neuropathy, HIV infection, leprosy, amputation, peripheral nerve injury pain, and stroke [2]. In this cross-sectional survey too, patients reported neuropathic pain of various etiology with varying severity and duration. Diabetic neuropathy was a significant contributor. Peripheral nerve injury, fibromyalgia, post-herpetic neuralgia, post-surgical pain, spinal cord injury, and nerve compression syndrome were reported as different causes of neuropathic pain in our study. Neuropathic pain is not alleviated by routine painkillers such as non-steroidal anti-inflammatory drugs (NSAIDs) and requires centrally acting intervention. The recommended options are TCAs, SSNRIs, gabapentinoids, tramadol, lidocaine, and capsaicin. Among these, gabapentinoids are considered the first-line treatment option for neuropathic pain. As neuropathic pain is a chronic condition with progressive severity, a single pharmacological intervention at times is not sufficient, and combination treatment becomes a necessity. The physician usually begins with a single agent and slowly progresses to combination therapy. This phenomenon was observed in our study too. Single-agent use, such as pregabalin or gabapentin, was higher at the first consultations. In contrast, at the follow-up visits, patients received combination therapy with either pregabalin + nortriptyline, gabapentin + nortriptyline, or pregabalin with duloxetine. The use of the pregabalin with duloxetine combination was the highest among all combinations. More than 30% of patients were prescribed this combination at the first visit and follow-up. This combination was prescribed to both male and female patients and was given to patients presenting moderate to severe pain intensity. Neuropathic pain is rarely mild and usually poses moderate to severe intensity, affecting daily functions [17,18].

In this study, too, patients presenting mild pain were very few compared to the patients presenting moderate to severe pain, making gabapentinoid-based combination therapy the most preferred treatment in the patients. Pregabalin with nortriptyline was the second preferred combination, followed by pregabalin with nortriptyline in patients with moderate to severe neuropathic pain. Although pregabalin with duloxetine was prescribed in more patients, pregabalin with nortriptyline demonstrated a slightly higher improvement rate of 85.32% vs. 83.78% in the pregabalin-duloxetine group. Almost all patients reported improvement in symptoms upon treatment. Few patients required a change in prescription at follow-up either due to no satisfactory efficacy or tolerance issues reported by patients. Approximately 20% of patients had a prescription change due to tolerance issues, and approximately 30% had a change of prescription due to lack of satisfactory efficacy. Pregabalin with duloxetine remained the predominant treatment option used by the treating clinician in most patients.

The drug utilization of the various pharmacological agents was in line with the national consensus statements published in 2018 [13]. However, our survey could not collect the doses prescribed by the clinicians. There have been some discussions about lowering the doses of the combination treatment to improve tolerance and treatment compliance [19], which could not be verified in this survey.

Opioids are not considered to be a first choice because of adverse drug reactions and their abuse and addictive potential [20]. For the treatment of neuropathic pain, there is strong evidence of the clinical use of gabapentinoids, or GABA-mimetic antiepileptic drugs [21]. Gabapentinoids, such as gabapentin and pregabalin, are first- and second-generation alpha-2 delta (α2δ) inhibitor ligands, respectively, and both are approved for use as adjunctive therapy in pain control. They exert analgesic activity by inhibiting injury-induced spinal neuronal excitability via the α2δ subunit of pre-synaptic calcium channels in the spinal cord. Gabapentin is effective in the treatment of post-herpetic neuralgia, diabetic neuropathy, trigeminal neuralgia, and pain syndromes following spinal cord injury, and also for deep tissue pain and hyperalgesia [22]. Globally, Gabapentin was first approved for use as an adjunct treatment for partial epileptic seizures in adults and children in 1993 and then for the treatment of chronic pain, in particular neuropathic pain syndromes [23,24]. 

Gabapentinoids inhibit the joint action of voltage-gated calcium channel (VGCC) α2δ subunits in conjunction with the NMDA receptor, with subsequent downregulation of VGCC expression and excitatory neurotransmitter release. This possibly results in the efficacy of gabapentinoids in the management of neuropathic pain. Gabapentinoids also facilitate slow-wave sleep, a relatively rare phenomenon among central nervous system-acting agents, which is also thought to explain some of the therapeutic benefits of the class in conditions such as fibromyalgia. There are reported over 50 million prescriptions per year in the USA alone. The gabapentinoids are safe and possess a low risk of misuse, abuse, and dependence [25-27].

The combination of gabapentinoids with the first-line TCA antidepressants amitriptyline and nortriptyline and SSNRI antidepressants duloxetine and venlafaxine is recommended in alleviating neuropathic pain [28,29]. In a systematic review and meta-analysis of five randomized clinical trials (RCTs), in patients with painful diabetic peripheral neuropathy, the combination of gabapentinoids with TCAs or SSNRIs was associated with a more significant reduction in pain as compared with monotherapy. Mean pain reduction was more significant for combination therapy than monotherapy (mean deviation (MD) - 0.39; 95% CI - 0.67 to - 0.12; p = 0.005). Similarly, there was an improvement in ≥ 30% reduction in average pain (relative risk (RR) 1.16; 95% CI 1.07-1.26; p < 0.01) with combination therapy. When comparing combination therapy versus gabapentinoid monotherapy, there was a significant reduction in average pain (MD - 0.61; 95% CI - 0.85 to - 0.37; p < 0.01) with combination therapy [30]. Our results show a similar trend wherein combination treatment of gabapentinoids with nortriptyline or duloxetine was more effective in pain relief compared to gabapentin or pregabalin when given alone.

Thus, the treatment for neuropathic pain is predominated by gabapentinoid-based pharmacotherapy, as reported in the literature [31,32]. More than 50 studies are reported in the literature with gabapentinoids used in various types of neuropathic pain. In a systematic meta-analysis, the efficacy and safety outcomes are reported in 15 studies with pregabalin and six studies with gabapentin. The pooled results showed that the pregabalin and gabapentin groups were significantly better than the placebo group, and the proportion of participants who achieved at least 30% pain reduction has been reported in these studies. Withdrawals due to lack of efficacy occurred in significantly fewer patients (3%) taking pregabalin. The reported adverse events were related to cognition/coordination, dizziness, somnolence, ataxia, amnesia, abnormal gait, incoordination, and asthenia [25,33]. In our study, the types of adverse effects of the treatment were not captured. However, the tolerance issues prompted a change in prescription in around 20% of the patients at the follow-up visit. The recommended first-line drugs, such as antidepressants TCA, SSNRI, and anticonvulsants acting at calcium channels (pregabalin and gabapentin), were used in our survey by the clinicians as recommended. The treatment of neuropathic pain is challenging due to its heterogeneous etiologies, lack of objective diagnostic tools, and resistance to classical analgesic drugs [12]. However, gabapentinoid-based treatment in combination with a tricyclic antidepressant or SSRI is useful in providing pain relief in patients and helps to improve the QoL.

## Conclusions

Neuropathic pain is a chronic and debilitating condition encountered in daily clinical practice. Gabapentinoid-based treatments combined with tricyclic antidepressants or SSRI agents provide rapid relief from pain. This DUS has shown its extensive use in alleviating neuropathic pain in the Indian neurology practice. 
